# Prevalence and patient-rated relevance of complexity factors in medication regimens of community-dwelling patients with polypharmacy

**DOI:** 10.1007/s00228-022-03314-1

**Published:** 2022-04-27

**Authors:** Viktoria S. Wurmbach, Steffen J. Schmidt, Anette Lampert, Simone Bernard, Andreas D. Meid, Eduard Frick, Michael Metzner, Stefan Wilm, Achim Mortsiefer, Bettina Bücker, Attila Altiner, Lisa Sparenberg, Joachim Szecsenyi, Frank Peters-Klimm, Petra Kaufmann-Kolle, Petra A. Thürmann, Walter E. Haefeli, Hanna M. Seidling

**Affiliations:** 1 Department of Clinical Pharmacology and Pharmacoepidemiology, Heidelberg University Hospital, Im Neuenheimer Feld 410, 69120 Heidelberg, Germany; 2 Cooperation Unit Clinical Pharmacy, Heidelberg University Hospital, Heidelberg, Germany; 3 Department of Clinical Pharmacology, School of Medicine, Faculty of Health, Witten/Herdecke University, Witten, Germany; 4 Institute of General Practice (ifam), Centre for Health and Society (chs), Medical Faculty, Heinrich Heine University Düsseldorf, Düsseldorf, Germany; 5 Professorship of Primary Care, Faculty of Health, Witten/Herdecke University, Witten, Germany; 6 Institute of General Practice, Rostock University Medical Center, Rostock, Germany; 7 Department of General Practice and Health Services Research, Heidelberg University Hospital, Heidelberg, Germany; 8 aQua-Institute for Applied Quality Improvement and Research in Health Care, Goettingen, Germany; 9 Philipp Klee-Institute for Clinical Pharmacology, HELIOS University Clinic Wuppertal, Wuppertal, Germany

**Keywords:** Polypharmacy, Medication administration, Medication regimen complexity, Patient-centered care, Shared decision-making, Adherence

## Abstract

**Purpose:**

To describe the prevalence of complexity factors in the medication regimens of community-dwelling patients with more than five drugs and to evaluate the relevance of these factors for individual patients.

**Methods:**

Data were derived from the HIOPP-6 trial, a controlled study conducted in 9 general practices which evaluated an electronic tool to detect and reduce complexity of drug treatment. The prevalence of complexity factors was based on the results of the automated analysis of 139 patients’ medication data. The relevance assessment was based on the patients’ rating of each factor in an interview (48 patients included for analysis).

**Results:**

A median of 5 (range 0–21) complexity factors per medication regimen were detected and at least one factor was observed in 131 of 139 patients. Almost half of these patients found no complexity factor in their medication regimen relevant.

**Conclusion:**

In most medication regimens, complexity factors could be identified automatically, yet less than 15% of factors were indeed relevant for patients as judged by themselves. When assessing complexity of medication regimens, one should especially consider factors that are both particularly frequent and often challenging for patients, such as use of inhalers or tablet splitting.

**Trial registration:**

The HIOPP-6 trial was registered retrospectively on May 17, 2021, in the German Clinical Trials register under DRKS-ID DRKS00025257.

**Supplementary information:**

The online version contains supplementary material available at 10.1007/s00228-022-03314-1.

## Introduction

Various complexity factors have already been identified in the literature that might increase complexity of drug treatment and, thus, make it potentially difficult for patients to administer their drugs correctly [1]. Most studies primarily identified single complexity factors or measured the level of medication regimen complexity by using a score such as the Medication Regimen Complexity Index (MRCI) [2, 3]. However, the prevalence of different complexity factors in the medication regimens and, thus, the distribution of complexity in distinct medication regimens have rarely been described. In addition, when assessing the complexity of a patient’s drug treatment, the patient’s perspective is usually not considered even though it ultimately determines the consequences of this complexity. In fact, a complexity factor might not be equally burdensome for all patients. For example, while drug administration at lunchtime is considered a complexity factor and has been associated with increased risk of nonadherence, this might not be true for all patients [4]. Hence, an adaptation of the dosing schedule might only be beneficial for patients that classify drug administration at lunchtime as inconvenient, error-prone, or requiring special preventive action.

To integrate the patient’s assessment of prevalent complexity, we have previously developed an electronic tool that combines an automated analysis of complexity of drug treatment with an individual assessment of the relevance of each complexity factor for the respective patient [5]. The electronic tool considers 38 complexity factors that can be identified automatically in structured medication data and can be assigned to one of the following categories: dosage form, dosage scheme, process characteristics, product characteristics, and additional instructions. Additional 14 rather generic complexity factors (e.g. an intricate packaging) were addressed by specific questions. Subsequently, standardized key questions were used to evaluate the relevance of the identified complexity factors for the individual patient. Thus, individualized optimization measures that eliminate a complexity factor or mitigate its impact can be proposed based on patient answers to the key question. The tool has been evaluated in an explorative, three-arm, controlled study (HIOPP-6) with 155 patients and revealed that an automated assessment of complexity of drug treatment that incorporated key questions led to significantly more recommendations that patients perceive helpful than an automated analysis alone [6].

The aim of this work is to give an overview of the frequency of the identification of complexity factors in a general practice population based on an automated analysis of structured medication data and to evaluate which of these complexity factors indeed pose a problem for patients.

## Methods

### Data collection

The analysis is based on data from the HIOPP-6 trial and considers data collected in all three study groups: In the first group, the electronic tool (automated and personalized analysis of complexity, intervention group) was used to analyze and reduce complexity of drug treatment, in a second group an exclusively automated analysis was conducted, and a third group formed the control group, representing routine care [6]. Patients were recruited non-consecutively by a total of nine general practitioners (three per group). For inclusion, patients had to take more than five drugs regularly (polypharmacy), to be at least 18 years old, and to give written informed consent. Patients’ sociodemographic data were collected using a paper-based questionnaire (including age, education, and prior medical knowledge).

### Prevalence of complexity factors in all patients

The medication of all patients was automatically searched for complexity factors with the electronic tool (retrospectively in the case of the control group). Thus, the evaluation of the prevalence of complexity factors in medication data is based on the automated analysis by the electronic tool. In all three groups, the patients’ medication at the time of study inclusion (i.e. prior to receiving the intervention in the first and second group) was used in this analysis. The complexity factor total number of drugs, defined as the use of more than five drugs, was not considered in the evaluation of prevalence because polypharmacy was one of the inclusion criteria.

### Relevance of the complexity factors in patients included in the intervention group

The relevance of the complexity factors identified automatically was assessed using key questions (personalization of the automated analysis) — however, only in the intervention group. These questions were proposed by the electronic tool every time a complexity factor was identified by the automated analysis and are intended to assess whether the patient indeed perceived the complexity factor as challenging (e.g. the key question “Do you find it difficult to split your tablets consistently into pieces that have the same size?” for the complexity factor tablet splitting and the key question “This drug should be used once a week. Is it difficult for you to use this drug always at the same day of the week?” for the complexity factor once weekly administration) [5]. If the patient indicated to experience any difficulties, the complexity factor was considered to be relevant for the patient in the analysis. Each patient of the intervention group was asked eight additional questions to evaluate the relevance of 14 complexity factors that could not be assessed automatically (non-automated evaluation; e.g. the question “Many patients find it difficult to remove their drugs from the packaging. Do you have any difficulties with the packaging of one of your drugs?” to address the complexity factor intricate packaging).

### Data analysis

The prevalence of complexity factors and their relevance for the patients were analyzed descriptively. In order to derive which factors are likely to be of actual importance in everyday care, we analyzed which of the rather frequent factors and which of the very frequent factors were considered most often relevant by patients. We assumed factors to be rather frequent if they occurred at least as often as the median (third and fourth quartile) in the intervention group, and factors to be very frequent if they occurred more often than the 3rd quartile. Values of categorical data are given as relative and absolute frequencies.

## Results

### Prevalence of complexity factors

Our study enrolled a total of 155 patients, 139 of these patients were included for the analysis of the complexity factors (Table 1).

**Table 1:** Sociodemographic data of the general practice population

	**Prevalence** **(*n* = 139)**	**Relevance** **(*n* = 48)**
**Mean age [years]** ± **SD (range)**	71.7 ± 11.3 (29–89) (missing answers: 3 patients)	71.7 ± 10.9 (33–89) (missing answer: 1 patient)
**Percentage of female patients [%]**	47.5 (missing answer: 1 patient)	43.8
**Mean number of drugs per patient ± SD**	9.4 (± 2.5)	10.0 (± 2.5)
**Education [%]**		
**- No graduation**	5.0	4.2
**- Lower secondary**	57.6	62.5
**- Secondary**	17.3	12.5
**- High school**	15.1	16.7
**- Other**	3.6	2.1
**- Missing answers [n]**	2	1
**Medical knowledge [%]**	5.8 (missing answers: 9 patients)	8.3
**Mean duration of treatment at respective general practice** ± **SD [years] (range)**	13.3 ± 12.3 (0.2–45) (missing answers: 8 patients)	12.4 ± 11.9 (0.5–45) (missing answers: 3 patients)

*SD* standard deviation

A total of 865 complexity factors were identified automatically in all three study groups combined and a median of 5 (range: 0–21) complexity factors were found per medication regimen. Indeed, 131 of 139 patients (94.2%) had at least one complexity factor in their medication regimen. The majority of complexity factors identified were related to the dosage scheme (59.8%; 517/865), and only few factors detected were associated to additional instructions (2.2%; 19/865) or the product (3.4%; 29/865) (Table 2).

**Table 2:** Prevalence of identification of complexity factors (automated identification) and their relevance for patients identified by key questions (personalization of automated detection)

**Complexity factor**	**Relative frequency of complexity factors [%]** **(absolute frequency); *n* = 865**	**Percentage of patients with complexity factor in medication regimen [%] (absolute frequency); *n* = 139**	**Percentage of each complexity factor being relevant for patients [%] (absolute frequency)**	**Percentage of patients having difficulties with distinct complexity factor [%] (absolute frequency)**
**Dosage form (162 complexity factors identified)**				
Injection devices (prefilled)^**^	6.8 (59)	25.2 (35)	15.0 (3/20)	23.1 (3/13)
Inhalers^**^	6.2 (54)	23.7 (33)	52.9 (9/17)	50.0 (5/10)
Metered dose inhaler	3.2 (28)	17.3 (24)	66.7 (4/6)	66.7 (4/6)
Elpenhaler	n/i	n/i	n/i	n/i
Nebulisers	n/i	n/i	n/i	n/i
Capsule-based inhalers	0.6(5)	3.6 (5)	100.0 (1/1)	100.0 (1/1)
Other inhalers	2.4 (21)	14.4 (20)	40.0 (4/10)	44.4 (4/9)
Liquid oral dosage forms^*^	3.0 (26)	18 (25)	0 (0/11)	0 (0/11)
With measuring device	1.0 (9)	6.5 (9)	0 (0/4)	0 (0/4)
Dry syrup	n/i	n/i	n/i	n/i
Drops	2.0 (17)	12.2 (17)	0 (0/7)	0 (0/7)
Injection devices (non-prefilled)^*^	0.8 (7)	5.0 (7)	20.0 (1/5)	20.0 (1/5)
**Dermatological preparations (prescription-only)**	0.7 (6)	4.3 (6)	0 (0/2)	0 (0/2)
**Ophthalmic preparations**	0.6 (5)	2.2 (3)	n/i	n/i
Drops	0.6 (5)	2.2 (3)	n/i	n/i
Ointment/creme/gel	n/i	n/i	n/i	n/i
**Transdermal patches**	0.3 (3)	2.2 (3)	n/i	n/i
**Nasal preparations (prescription-only)**	0.2 (2)	1.4 (2)	n/i	n/i
**Solid dosage forms for oropharyngeal use**	n/i	n/i	n/i	n/i
**Liquid dosage forms for oropharyngeal use**	n/i	n/i	n/i	n/i
**Rectal preparations**	n/i	n/i	n/i	n/i
**Otological preparations**	n/i	n/i	n/i	n/i
**Vaginal preparations**	n/i	n/i	n/i	n/i
**Dosage scheme (517 complexity factors identified)**				
Tablet splitting^**^	14.2 (123)	56.1 (78)	19.1 (9/47)	25.8 (8/31)
Administration at lunch time^**^	11.1 (96)	42.4 (59)	12.5 (6/48)	17.2 (5/29)
Medication on demand^**^	10.2 (88)	37.4 (52)	4.9 (2/41)	9.5 (2/21)
Only one drug at one specific point in time^**a^	8.0 (69)	49.6 (69)	12.5 (3/24)	12.5 (3/24)
Administration more than two times daily^**^	3.9 (34)	24.5 (34)	6.7 (1/15)	6.7 (1/15)
Use of multiple doses concurrently^*^	3.5 (30)	17.3 (24)	0 (0/10)	0 (0/10)
The same active ingredient in different preparations^*^	3.4 (29)	17.3 (24)	0 (0/9)	0 (0/8)
Different doses of the same active ingredient at different times of day^*^	2.9 (25)	17.3 (24)	0 (0/10)	0 (0/10)
Once weekly administration^*^	1.3 (11)	6.5 (9)	25.0 (1/4)	25.0 (1/4)
**Variable dosing**	0.6 (5)	2.9 (4)	n/i	n/i
**Administration every two days or less frequently**	0.5 (4)	2.9 (4)	0 (0/2)	0 (0/2)
**Occasional, episodic drug treatment**	0.3 (3)	2.2 (3)	100.0 (1/1)	100.0 (1/1)
**Total number of drugs**^**a**^	n/a^b^	n/a^b^	8.5 (4/47^c^)	8.5 (4/47^c^)
**Fixed dosing interval**	n/i	n/i	n/i	n/i
**Additional instructions (19 complexity factors identified)**				
Meal-dependent administration^*^	1.4 (12)	7.2 (10)	33.3 (3/9)	37.5 (3/8)
Administration at fixed times of the day^*^	0.8 (7)	2.2 (3)	20.0 (1/5)	50.0 (1/2)
**Crushing tablets**	n/i	n/i	n/i	n/i
**Decreasing doses**	n/i	n/i	n/i	n/i
**Disintegrating tablets, capsules and powders**	n/i	n/i	n/i	n/i
**Increasing doses**	n/i	n/i	n/i	n/i
**Intake with advised liquid (or food)**	n/i	n/i	n/i	n/i
**Opening capsules**	n/i	n/i	n/i	n/i
**Product characteristics (29 complexity factors identified)**				
Potentially patient-unfriendly nature of liquid oral dosage forms^**^	3.4 (29)	18.7 (26)	15.4 (2/13)	16.7 (2/12)
**Process characteristics (138 complexity factors identified)**				
Potentially increased need for training in dosage form use^**^	15.4 (133)	51.8 (72)	5.1 (2/39)^d^	10.0 (2/20)^d^
Complex measurements (self-performed)^*^	0.6 (5)	3.6 (5)	33.3 (1/3)	33.3 (1/3)

*n/i* not identified, *n/a* not applicable

^*^Complexity factors that are rather frequent (identified at least three times in the intervention group); ^**^Complexity factors that are very frequent (identified at least 13 times in the intervention group)

^a^Complexity factor could only be identified once in each medication regimen

^b^As the complexity factor “Total number of drugs”, defined as the regular use of more than 5 drugs, was an inclusion criterion, the factor was not considered for the evaluation of the prevalence of complexity factors

^c^Due to an undetected technical error the complexity factor was not identified in one medication schedule by the tool and, thus, the relevance could not be assessed

^d^Patients were asked whether they already received any training. Only those who did not, were asked the key question on relevance

The most common complexity factor was a potentially increased need for training in the use of the dosage forms. Tablet splitting was the second most frequently detected complexity factor with more than half of all patients having this complexity factor in their medication regimen. Administration at lunchtime and medication on demand represented about one-tenth of all complexity factors identified in the analysis. Injection devices (prefilled) and inhalers were the most common potentially complex dosage forms that were identified. Many other potential factors were never or only rarely detected (e.g. vaginal or otological preparations).

### Relevance of complexity factors for patients

The intervention cohort consisted of 52 patients that fulfilled the inclusion criteria. As four patients could not be considered due to missing data (incorrect use of the electronic tool), 48 patients (Table 1) were included in the evaluation of the relevance of the complexity factors. The prevalences of the complexity factors were largely balanced in the intervention group and the two other groups, so it may be reasonably assumed that the results are transferable also to the entire cohort (Online Resource 1).

More than half of these patients (56.3%; 27/48) confirmed that they actually had difficulties related to at least one of the automatically identified complexity factors and an even larger proportion of patients (72.9%; 35/48) reported that one of the complexity factors that could not be assessed automatically was relevant to them (non-automated evaluation).

**Fig. 1:**
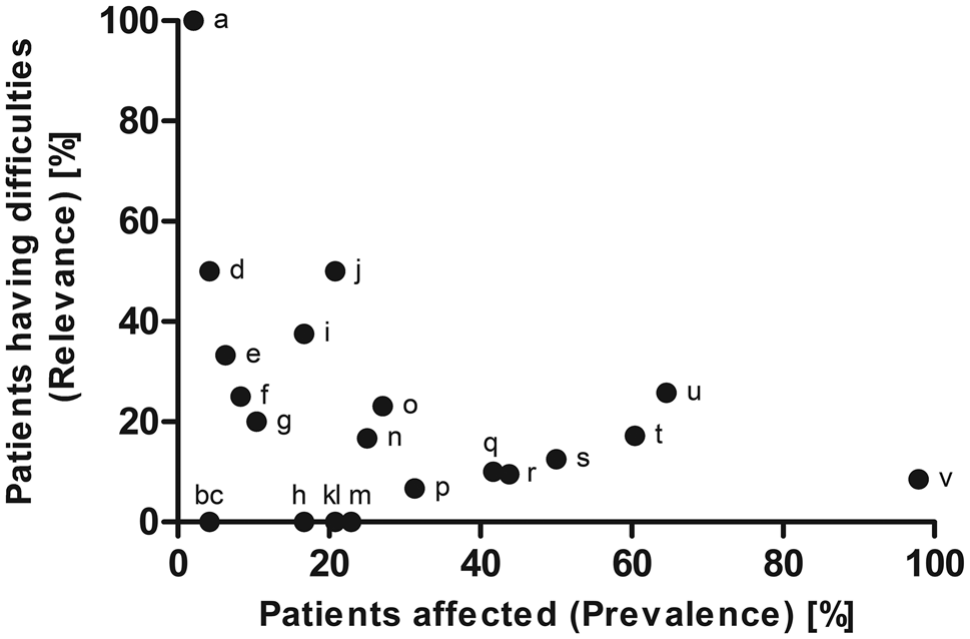
Percentage of patients for whom a distinct complexity factor was identified in the medication regimen (prevalence) and percentage of patients that indeed experience difficulties by the complexity factor (relevance) in the intervention group (*N* = 48); only complexity factors identified at least once in the intervention group considered. (**a**) Occasional, episodic drug treatment. (**b**) Dermatological preparations (prescription-only). (**c**) Administration every two days or less frequently. (**d**) Administration at fixed times of the day. (**e**) Complex measurements (self-performed). (**f**) Once weekly administration. (**g**) Injection devices (non-prefilled). (**h**) The same active ingredient in different preparations. (**i**) Meal-dependent administration. (**j**) Inhalers. (**k**) Use of multiple doses concurrently. (**l**) Different doses of the same active ingredient at different times of day. (**m**) Liquid oral dosage forms. (**n**) Potentially patient-unfriendly nature of liquid oral dosage forms. (**o**) Injection devices (prefilled). (**p**) Administration more than two times daily. (**q**) Potentially increased need for training in dosage form use. (**r**) Medication on demand. (**s**) Only one drug at one specific point in time. (**t**) Administration at lunch time. (**u**) Tablet splitting. (**v**) Total number of drugs

Overall, only 12.8% (49/382) of the automatically identified complexity factors were indeed relevant for the patients according to the key questions. The use of an inhaler was difficult for half of the patients exposed to this factor (50.0%; 5/10). Tablet splitting, the most prevalent complexity factor in this cohort (based on the number of patients with the factor in their medication regimen), was indeed difficult for one-quarter of the respective patients (25.8%; 8/31), while the total number of drugs, a complexity factor that applied to all medication regimens in this study due to it being an inclusion criterion, only increased the difficulty of using their medicines for less than one-tenth of patients (8.5%; 4/47) (Fig. 1).

Finally, some complexity factors were not relevant for any patient (e.g. liquid oral dosage forms or use of multiple doses concurrently).

The answers to the questions for the non-automated evaluation showed that half of patients (50.0%; 24/48) do not use their medication schedule regularly (complexity factor no use of medication schedule). Almost a third of patients reported problems remembering the names, administration times, and dosages of their drugs. However, less than 5% of patients (4.2%; 2/48) experienced swallowing problems (Table 3).

**Table 3:** Relevance of complexity factors that were evaluated non-automated

**Complexity factors identified non-automatically**	**Percentage of patients having difficulties with a complexity factor (*n* = 48))**
**No use of medication schedule**	50.0 (24)
**Cognitive impairment**	29.2 (14)
**Intricate packaging**	12.5 (6)
**Physical impairment**	12.5 (6)
**Similar drug names/similar drug appearance**	10.4 (5)
**Changes in existing medication regimen, New prescription, frequent generic substitution, Changes in tablet color or shape, Hospital discharge**	10.4 (5)
**Swallowing difficulties**	4.2 (2)
**Diverse storage conditions**	0 (0)

### Estimated importance of complexity factors in daily care

In the intervention group, the complexity factors were identified a median of 2.5 times (IQR = 0–12.5); total number of drugs included for analysis). Among the factors identified at least three times in all patients (third and fourth quartiles), the most relevant factors for patients were inhalers (rated as relevant in 52.9% of cases; *N* = 9/17 patients), meal-dependent administration (33.3%; 3/9), complex measurements (self-performed) (33.3%; 1/3), once weekly administration (25.0%; 1/4), injection devices (non-prefilled) (20.0%; 1/5), administration at fixed times of the day (20.0%; 1/5) and tablet splitting (19.1%; 9/47). Considering only the very frequent factors (fourth quartile; identified at least 13 times), inhalers (52.9%; 9/17, tablet splitting (19.1%; 9/47), potentially patient-unfriendly nature of liquid oral dosage forms (15.4%; 2/13), injection devices (prefilled) (15.0%; 3/20), administration at lunch time (12.5%; 6/48), and only one drug at one specific point in time (12.5%; 3/24) were the most relevant factors.

## Discussion

The medication regimens of more than 90% of polypharmacy patients in this general practice population contained at least one complexity factor and half of the patients stated that they actually had difficulties due to at least one complexity factor. The complexity factors identified most often were a potentially increased need for training in dosage form use, tablet splitting, and administration at lunch time. Among those factors that were identified most frequently, inhalers, tablet spitting, and a potentially patient-unfriendly nature of liquid oral dosage forms were most often rated as relevant by patients.

Factors in the “dosage scheme” category most often contributed to the complexity of drug treatment. This is in accordance with the findings of Metz et al. who applied the MRCI to the medication regimens of HIV-infected patients and observed that the dosage scheme was responsible for about two-thirds of the identified complexity [7]. The most common complexity factors of the category “dosage scheme” were tablet splitting, administration at lunchtime, medication on demand, or only one drug at one specific point in time. Approximately one in six patients described administration at lunchtime as a relevant complexity factor, and about one-tenth of polypharmacy patients found it difficult to take medication on-demand or to administer only one drug at any given time. However, some complexity factors, such as occasional episodic treatment or once weekly administration, were particularly relevant for the patients, albeit only rarely detected in the medication regimens analyzed.

A potentially increased need for training in dosage form use was identified most often. As most dosage forms are potentially difficult for patients, automated identification of this complexity factor was based on the identification of these dosage forms in the medication regimen of patients — leading to the large number of identifications. In one-tenth of cases in which the complexity factor was identified, patients stated not to have received any training in the use of the respective dosage form, although training has been shown to prevent errors in the handling of for example inhalers [8].

Splitting tablets was the complexity factor identified the second most frequently—more than half of patients regularly split tablets. This number might be even higher when considering also patients that split their tablets to facilitate swallowing. Indeed, in a survey among patients in Germany, 67.2% of participants stated to currently split tablets [9]. Problems associated with splitting tablets have already been described in the literature [10] and also in this evaluation, one in four patients rated the splitting of at least one of their tablets as indeed difficult. In a survey with patients splitting tablets regularly, more than 70% of patients agreed that even scored tablets cannot be divided into equal parts. A comparable proportion of patients stated that it is sometimes difficult to break tablets [9]. Thus, it should be evaluated carefully, if splitting tablets is feasible for the individual patient, because it could be prevented in almost half of the cases [11].

Injectables (prefilled) and inhalers were the most frequently detected potentially complex dosage forms. Thereby, inhalers seem to be more difficult for patients than injectables, because half of these patients indeed had problems using their inhaler. Comparably large proportions were also found in the literature when difficulties with and errors in inhaler use were evaluated; depending on the type of inhaler, proportions of patients experiencing at least one error in inhaler administrations ranged from 50 to 100% [12]. For some complexity factors, our findings differ from previous results. For example, only 4.2% of patients reported to have swallowing difficulties concerning at least one of their drugs. In previous studies, around one-tenth and up to more than one-quarter of ambulatory patients reported to have swallowing problems with their current medication [13, 14]. However, a comparison of the results with those of previous studies is limited, because there is no standardized approach to measure complexity of drug treatment or to determine the relevance of factors potentially increasing complexity for individual patients.

To this day, the complexity of drug treatment is usually neglected in routine care, so that there is a constant risk of overloading patients with their drug treatment. The potential consequences of a complex drug treatment are nonadherence, hospitalizations, hospital readmissions, and adverse drug events [15–18]; even a correlation with a worse quality of life has recently been found in patients living with HIV [19]. Furthermore, it has been shown to better predict all-cause mortality than polypharmacy, but an association could only be demonstrated in men, participants aged 80 years or younger, and participants with a good cognition (measured by Mini-Mental State Examination with a score of at least 26) [20].

But the effect on patient outcomes of an intervention to reduce complexity has only rarely been considered [21, 22] and should be further investigated. However, several measures to simplify a regimen (reduction of drug burden, i.e. use of fixed-dose combinations or single-tablet regimens, or dosing frequency, i.e. an once-daily dosing, or a combination of these measures) have been shown to improve patient’s adherence in several studies, although a consistent positive effect on clinical outcomes could not be shown [23, 24]. Hence, several implications for patient care can be derived from this work. Complexity factors should be considered routinely by health professionals, especially those that are highly relevant for patients such as inhalers or tablet splitting, since up to half of patients find these administration steps difficult. Furthermore, these difficulties should be taken into account in the development of new drugs and in clinical trials to ensure that the use will not pose any difficulties to the target population [25]. But the use of an automated analysis of complexity of drug treatment alone might lead to an overestimation of patients’ difficulties, when the patient’s perspective is not considered. The results show that asking patients specific questions on well-known complexity factors already leads to the identification of a remarkable amount of difficulties experienced by patients when handling their drug treatment. Thus, the use of standardized question is an approach that could be implemented in routine care to assess patients’ perspective. Thereby, even simple measures, such as an adaptation of the prescribed dose or the dosing frequency [11] or training [26], could often reduce or at least mitigate complexity of drug treatment.

This work has several limitations. First, only a limited number of (mostly elderly) polypharmacy patients visiting a general practitioner were considered in this work and, thus, the distribution of complexity factors in other patient populations (e.g. patients on dialysis or with cognitive dysfunction) might vary. It might be assumed that the prevalence but also, most importantly, the relevance of individual factors varies depending on the prevalence of certain conditions such as dementia or Parkinson’s disease. Second, the analysis of complexity of drug treatment was conducted by the patients’ general practitioners and, hence, answers to the key questions were certainly subject to social desirability due to the longstanding relationship between patients and doctors for more than 12 years on average.

## Conclusion

In an automated analysis of complexity in drug treatment, a median of 5 complexity factors were found in the medication data of each patient in a general practice population taking more than five drugs regularly. Three complexity factors were identified the most frequently: A potentially increased need for training in dosage form use, tablet splitting, and administration at lunch time were most frequently identified. The personalization of the results by means of standardized key questions showed that more than half of the patients actually experience difficulties with at least one of these factors. Especially the use of inhalers, tablet splitting and a potentially patient-unfriendly nature of liquid oral dosage forms turned out to be both particularly frequent and relevant in this patient population. However, less than 15% of the factors identified in the automated analysis were indeed relevant for patients as judged by themselves.

## Supplementary Information

Below is the link to the electronic supplementary material.

Supplementary file1 (PDF 205 KB)

## Data Availability

The data that support the findings of this study are available from the corresponding author upon reasonable request.

## References

[1] Schmidt SJ, Wurmbach VS, Lampert A, Bernhard S, HIOPP-6 Consortium, Haefeli WE, Seidling HM, Thürmann PA (2020). Individual factors increasing complexity of drug treatment-a narrative review. Eur J Clin Pharmacol.

[2] Paquin AM, Zimmerman KM, Kostas TR, Pelletier L, Hwang A, Simone M, Skarf LM, Rudolph JL (2013). Complexity perplexity: a systematic review to describe the measurement of medication regimen complexity. Expert Opin Drug Saf.

[3] George J, Phun YT, Bailey MJ, Kong DC, Stewart K (2004). Development and validation of the medication regimen complexity index. Ann Pharmacother.

[4] Martin-Latry K, Cazaux J, Lafitte M, Couffinhal T (2014). Negative impact of physician prescribed drug dosing schedule requirements on patient adherence to cardiovascular drugs. Pharmacoepidemiol Drug Saf.

[5] Wurmbach VS, Schmidt SJ, Lampert A, Frick E, Metzner M, Bernard S, Thürmann PA, Wilm S, Mortsiefer A, Altiner A, Sparenberg L, Szecsenyi J, Peters-Klimm F, Kaufmann-Kolle P, Haefeli WE, Seidling HM (2020). Development of an algorithm to detect and reduce complexity of drug treatment and its technical realisation. BMC Med Inform Decis Mak.

[6] Wurmbach V, Seidling H, Haefeli WE (2021) Ergebnisbericht. Innovationsausschuss beim Gemeinsamen Bundesausschuss. https://innovationsfonds.g-ba.de/downloads/beschluss-dokumente/20/2020-04-03_HIOPP-6_Ergebnisbericht.pdf. Accessed 25 Feb 2022

[7] Metz KR, Fish DN, Hosokawa PW, Hirsch JD, Libby AM (2014). Patient-level medication regimen complexity in patients with HIV. Ann Pharmacother.

[8] Arora P, Kumar L, Vohra V, Sarin R, Jaiswal A, Puri MM, Rathee D, Chakraborty P (2014). Evaluating the technique of using inhalation device in COPD and bronchial asthma patients. Respir Med.

[9] Quinzler R, Szecsenyi J, Haefeli WE (2007). Tablet splitting: patients and physicians need better support. Eur J Clin Pharmacol.

[10] Freeman MK, White W, Iranikhah M (2012). Tablet splitting: a review of weight and content uniformity. Consult Pharm.

[11] Witticke D, Seidling HM, Lohmann K, Send AFJ, Haefeli WE (2013). Opportunities to reduce medication regimen complexity: a retrospective analysis of patients discharged from a university hospital in Germany. Drug saf.

[12] Chrystyn H, van der Palen J, Sharma R, Barnes N, Delafont B, Mahajan A, Thomas M (2017). Device errors in asthma and COPD: systematic literature review and meta-analysis. NPJ Prim Care Respir Med.

[13] Schiele JT, Quinzler R, Klimm HD, Pruszydlo MG, Haefeli WE (2013). Difficulties swallowing solid oral dosage forms in a general practice population: prevalence, causes, and relationship to dosage forms. Eur J Clin Pharmacol.

[14] Marquis J, Schneider MP, Payot V, Cordonier AC, Bugnon O, Hersberger KE, Arnet I (2013). Swallowing difficulties with oral drugs among polypharmacy patients attending community pharmacies. Int J Clin Pharm.

[15] Wimmer BC, Cross AJ, Jokanovic N, Wiese MD, George J, Johnell K, Diug B, Bell JS (2017). Clinical outcomes associated with medication regimen complexity in older people: a systematic review. J Am Geriatr Soc.

[16] Alves-Conceicao V, Rocha KSS, Silva FVN, Silva ROS, da Silva DT, Lyra-Jr DP (2018). Medication regimen complexity measured by MRCI: a systematic review to identify health outcomes. Ann Pharmacother.

[17] Alves-Conceicao V, Rocha KSS, Silva FVN, Silva ROS, Cerqueira-Santos S, Nunes MAP, Martins-Filho PRS, da Silva DT, Lyra-Jr DP (2020). Are clinical outcomes associated with medication regimen complexity? A systematic review and meta-analysis. Ann Pharmacother.

[18] Brysch EG, Cauthon KAB, Kalich BA, Sarbacker GB (2018). Medication regimen complexity index in the elderly in an outpatient setting: a literature review. Consult Pharm.

[19] Contreras-Macias E, Gutierrez-Pizarraya A, Robustillo-Cortes MA, Morillo-Verdugo R (2021) High level of medication regimen complexity index correlate with worse quality of life in people living with HIV. Rev Esp Quimioter 34:93–99. 10.37201/req/097.2020 10.37201/req/097.2020 PMC8019467 33499583

[20] Wimmer BC, Bell JS, Fastbom J, Wiese MD, Johnell K (2016). Medication regimen complexity and polypharmacy as factors associated with all-cause mortality in older people: a population-based cohort study. Ann Pharmacother.

[21] Stange D, Kriston L, von-Wolff A, Baehr M, Dartsch DC (2013) Reducing cardiovascular medication complexity in a German university hospital: effects of a structured pharmaceutical management intervention on adherence. J Manag Care Pharm 19:396–407. 10.18553/jmcp.2013.19.5.396 10.18553/jmcp.2013.19.5.396 PMC10437592 23697477

[22] Dugre N, Bell JS, Hopkins RE, Ilomäki J, Chen EYH, Corlis M (2021). Impact of medication regimen simplification on medication incidents in residential aged care: SIMPLER randomized controlled trial. J Clin Med.

[23] Elnaem MH, Irwan NA, Abubakar U, Syed Sulaiman SA, Elrggal ME, Cheema E (2020). Impact of medication regimen simplification on medication adherence and clinical outcomes in patients with long-term medical conditions. Patient Prefer Adherence.

[24] Huffman MD, Xavier D, Perel P (2017). Uses of polypills for cardiovascular disease and evidence to date. Lancet.

[25] Wahlich J, Orlu M, Mair A, Stegemann S, van Riet-Nales D (2019). Age-related medicine Pharmaceutics.

[26] Omori K, Kawamura T, Urata M, Matsuura M, Kusama M, Imamine R, Watarai A, Nakashima E, Umemura T, Hotta N (2017). Effect of re-coaching on self-injection of insulin in older diabetic patients - impact of cognitive impairment. Diabetes Res Clin Pract.

