# Correction to: Prevalence and patient‑rated relevance of complexity factors in medication regimens of community dwelling patients with polypharmacy

**DOI:** 10.1007/s00228-022-03342-x

**Published:** 2022-06-02

**Authors:** Viktoria S. Wurmbach, Steffen J. Schmidt, Anette Lampert, Simone Bernard, Andreas D. Meid, Eduard Frick, Michael Metzner, Stefan Wilm, Achim Mortsiefer, Bettina Bücker, Attila Altiner, Lisa Sparenberg, Joachim Szecsenyi, Frank Peters‑Klimm, Petra Kaufmann‑Kolle, Petra A. Thürmann, Walter E. Haefeli, Hanna M. Seidling

**Affiliations:** 1 Department of Clinical Pharmacology and Pharmacoepidemiology, Heidelberg University Hospital, Im Neuenheimer Feld 410, 69120 Heidelberg, Germany; 2 Cooperation Unit Clinical Pharmacy, Heidelberg University Hospital, Heidelberg, Germany; 3 Department of Clinical Pharmacology, School of Medicine, Faculty of Health, Witten/Herdecke University, Witten, Germany; 4 Institute of General Practice (ifam), Centre for Health and Society (chs), Medical Faculty, , Heinrich Heine University Düsseldorf, Düsseldorf, Germany; 5 Professorship of Primary Care, Faculty of Health, Witten/Herdecke University, Witten, Germany; 6 Institute of General Practice, Rostock University Medical Center, Rostock, Germany; 7 Department of General Practice and Health Services Research, Heidelberg University Hospital, Heidelberg, Germany; 8 aQua-Institute for Applied Quality Improvement and Research in Health Care, Goettingen, Germany; 9 Philipp Klee-Institute for Clinical Pharmacology, HELIOS University Clinic Wuppertal, Wuppertal, Germany

## Correction to: European Journal of Clinical Pharmacology https://doi.org/10.1007/s00228-022-03314-1

The assigned affiliations did not match the number of affiliations in the published proof.

The Original article has been corrected.

