# Supplementary material for: Prevalence and patient-rated relevance of complexity factors in medication regimens of community-dwelling patients with polypharmacy
Source: Eur J Clin Pharmacol. 2022 Apr 27;78(7):1127–36. doi: 10.1007/s00228-022-03314-1 (PMC9184426; doi:10.1007/s00228-022-03314-1)
Supplement: Supplementary file 1 — Supplementary file1 (PDF 205 KB) [file 228_2022_3314_MOESM1_ESM.pdf]

Prevalence and patient-rated relevance of complexity factors in medication regimens of community-dwelling patients with polypharmacy

Viktoria S. Wurmbach[1,2*]; Steffen J. Schmidt[3*]; Anette Lampert[1,2]; Simone Bernard[3]; Andreas D. Meid[1]; Eduard Frick[1]; Michael Metzner[1]; Stefan Wilm[4]; Achim Mortsiefer[5,6]; Bettina Bücker[4]; Attila Altiner[7]; Lisa Sparenberg[7]; Joachim Szecsenyi[8]; Frank Peters-Klimm[8]; Petra Kaufmann-Kolle[9]; Petra A. Thürmann[3,10]; Walter E. Haefeli[1,2]; Hanna M. Seidling[1,2]

[*] *both authors contributed equally to the work*

[1]Department of Clinical Pharmacology and Pharmacoepidemiology, Heidelberg University Hospital, Heidelberg, Germany; [2]Cooperation Unit Clinical Pharmacy, Heidelberg University Hospital, Heidelberg, Germany; [3]Department of Clinical Pharmacology, School of Medicine, Faculty of Health, Witten/Herdecke University, Witten, Germany; [4]Institute of General Practice (ifam), Centre for Health and Society (chs), Medical Faculty, Heinrich Heine University Düsseldorf, Düsseldorf, Germany; [5]Institute of General Practice (ifam), Centre for Health and Society (chs), Medical Faculty, Heinrich Heine University Düsseldorf, Düsseldorf, Germany (affiliation during study conduct); [6]Professorship of Primary Care, Faculty of Health, Witten/Herdecke University, Witten, Germany (current affiliation); [7]Institute of General Practice, Rostock University Medical Center, Rostock, Germany; [8]Department of General Practice and Health Services Research, Heidelberg University Hospital, Heidelberg, Germany; [9]aQua-Institute for Applied Quality Improvement and Research in Health Care, Goettingen, Germany; [10]Philipp Klee-Institute for Clinical Pharmacology, HELIOS University Clinic Wuppertal, Wuppertal, Germany

Corresponding author:
Prof. Dr. sc. hum. Hanna M. Seidling
Heidelberg University Hospital
Department of Clinical Pharmacology and Pharmacoepidemiology
Cooperation Unit Clinical Pharmacy
Im Neuenheimer Feld 410
69120 Heidelberg
Germany
Tel +49 6221/56-38736
Fax +49 6221/56-4642
Email hanna.seidling@med.uni-heidelberg.de

Figure S1

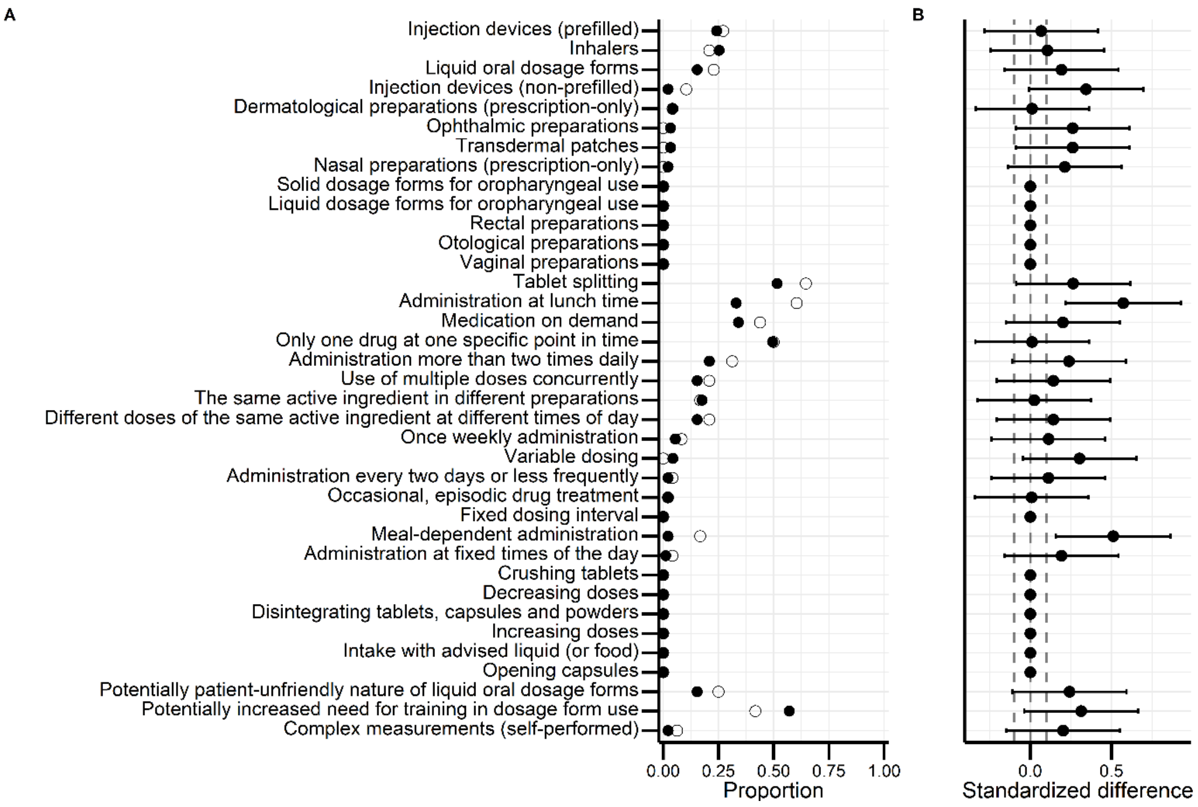

A: Proportion of patients in the intervention group and the other two groups for whom a distinct complexity factor was identified at least once in the medication regimen (open circle: intervention group, filled circle: the other two groups); B: Standardized difference of proportions of intervention group and the other two groups (with 95 % confidence limits indicated by error bars). The (strict) range for balanced groups (by convention) between -0.1 and 0.1 is highlighted by vertical dashed lines.
